# Core Components of Abscisic Acid Signaling and Their Post-translational Modification

**DOI:** 10.3389/fpls.2022.895698

**Published:** 2022-05-30

**Authors:** Junsub Lim, Chae Woo Lim, Sung Chul Lee

**Affiliations:** Department of Life Science (BK21 Program), Chung-Ang University, Seoul, South Korea

**Keywords:** abscisic acid, core component of ABA signaling, phosphorylation, post-translational modification, ubiquitination

## Abstract

Abscisic acid (ABA) is a major phytohormone that regulates plant growth, development, and abiotic/biotic stress responses. Under stress, ABA is synthesized in various plant organs, and it plays roles in diverse adaptive processes, including seed dormancy, growth inhibition, and leaf senescence, by modulating stomatal closure and gene expression. ABA receptor, clade A protein phosphatase 2C (PP2C), and SNF1-related protein kinase 2 (SnRK2) proteins have been identified as core components of ABA signaling, which is initiated *via* perception of ABA with receptor and subsequent activation or inactivation by phosphorylation/dephosphorylation. The findings of several recent studies have established that the post-translational modification of these components, including phosphorylation and ubiquitination/deubiquitination, play important roles in regulating their activity and stability. In this review, we discuss the functions of the core components of ABA signaling and the regulation of their activities *via* post-translational modification under normal and stress conditions.

## Introduction

Abscisic acid (ABA) is a major hormone that regulates growth, development, and responses to abiotic/biotic stress throughout the life cycle of plants. Under environmental conditions perceived as unsuitable for plant growth, ABA helps maintain seed dormancy and inhibits germination, whereas during the post-germination stage, this hormone inhibits growth to conserve energy (Chinnusamy et al., 2008; Raghavendra et al., 2010). In addition, ABA plays key roles in stomatal opening and closure, thereby contributing to the regulation of water loss under drought stress (Melotto et al., 2006; Hirayama and Shinozaki, 2007; Cutler et al., 2010; Finkelstein, 2013).

The core components of ABA signaling, along with their roles and regulatory mechanisms, are well known. As ABA receptors, pyrabactin resistance 1 (PYR1)/PYR1-like (PYL)/regulatory components of the ABA receptor (RCAR) family bind ABA in response to abiotic stress and positively regulate ABA signaling (Ma et al., 2009; Park et al., 2009; Santiago et al., 2009; Gonzalez-Guzman et al., 2012; Antoni et al., 2013). Under normal conditions, clade A protein phosphatase 2Cs (PP2Cs) play roles as negative regulator of ABA signaling by binding to subclass III sucrose nonfermenting 1 (SNF1)-related protein kinase 2 (SnRK2) proteins, and subsequently these interactions inactivate downstream ABA signaling factors and inhibit ABA responses to facilitate growth and development (Mustilli et al., 2002; Yoshida et al., 2006a; Fujii et al., 2007; Umezawa et al., 2009; Vlad et al., 2009). Under stress conditions, ABA receptors interact with PP2Cs and SnRK2s are released from PP2Cs-mediated inhibition, leading to autophosphorylation or phosphorylation by other kinases, whereupon the activated SnRK2s phosphorylate downstream targets, including transcription factors and channel proteins, thereby facilitating abiotic stress responses (Fujii and Zhu, 2009; Geiger et al., 2009; Yin et al., 2009; Nishimura et al., 2010; Ng et al., 2011; Xue et al., 2011; Brandt et al., 2012; Soon et al., 2012).

The different components of ABA signaling are modulated to varying extents by post-translational modification (Table 1), with the ubiquitination/deubiquitination and phosphorylation of these components being generally well studied. Ubiquitination of ABA core components can influence ABA signaling and responses both positively and negatively (Coego et al., 2021). Deubiquitination mediated by members of the UBIQUITIN-SPECIFIC PROTEASE (UBP) protein family affects a range of plant physiological processes, including growth, development, immunity, and stress response (Zhou et al., 2017). However, the biochemical mechanisms underlying the involvement of UBPs in ABA signaling have yet to be sufficiently elucidated. A further major category of post-translational modification is phosphorylation; ABA receptors, PP2Cs, and SnRK2s can be positively or negatively regulated by phosphorylation. For example, SnRK2s can undergo autophosphorylation and activation (Yang et al., 2017; Chen et al., 2020). In this review, we focus on the roles of ABA, core ABA signaling pathways, and post-transcriptional regulation of ABA signaling components in plants.

**Table 1:** Post-translational regulation factors of ABA core components in *Arabidopsis*.

Post-translational regulation	Enzyme	Targets	References
Ubiquitination	RSL1	PYR1/PYL4	Bueso et al., 2014
Ubiquitination	CRL4^DDA1^	PYL8	Irigoyen et al., 2014
Ubiquitination	RFA1/RFA4	PYR1/PYL4	Fernandez et al., 2020
Ubiquitination	PUB12/13	ABI1	Kong et al., 2015
Ubiquitination	CRL3^BPM3/5^	PP2CA, ABI1, HAB1	Julian et al., 2019
Ubiquitination	AIRP3	ABI1	Pan et al., 2020
Ubiquitination	PIR1/2	PP2CA	Baek et al., 2019b
Ubiquitination	RGLG1/5	PP2CA, ABI2, HAB2	Wu et al., 2016
Endosomal degradation	FYVE1/FREE1	PYR1/PYL4	Belda-Palazon et al., 2016
Endosomal degradation	VPS23A	PYR1/PYL4	Yu et al., 2016
Endosomal degradation	ALIX	PYL4	Garcia-Leon et al., 2019
Phosphorylation	TOR	PYL1/PYL4	Wang et al., 2018a
Phosphorylation	AEL	PYR1/PYL1	Chen et al., 2018
Phosphorylation	CEPR2	PYL1/PYL2/PYL4	Yu et al., 2019
Phosphorylation	CARK1	PYR1/PYL1/PYL2/PYL3/PYL8	Zhang et al., 2018; Li et al., 2019
Phosphorylation	PR5K2	ABI1/2	Baek et al., 2019a
Phosphorylation	BIN2	SnRK2.2/2.3	Cai et al., 2014
Phosphorylation	RAF10	SnRK2.2/2.3/2.6	Nguyen et al., 2019

## The Role of ABA in Abiotic Stress Responses

### Regulation of Stomatal Closure

In response to drought stress, ABA induces stomatal closure to reduce transpirational water loss (Bauer et al., 2013). ABA modulates stomatal movement *via* both Ca^2+^-dependent and -independent pathways. In the former, ABA induces Ca^2+^ channel opening to facilitate the influx of calcium ions, in response to which upregulated reactive oxygen species levels and inositol-1-4-5-triphosphate contribute to increases in cytosolic Ca^2+^ content (Lee et al., 1996; Pei et al., 2000; Murata et al., 2001; Mustilli et al., 2002), which in turn modulates multiple signal transduction. Calcium-dependent protein kinases (CPKs) 3/4/6/10/11 phosphorylate and activate slow-type anion efflux channels, including SLOW ANION CHANNEL-ASSOCIATED 1 (SLAC1) and SLAC1 HOMOLOG 3 (SLAH3; Mori et al., 2006; Zhu et al., 2007; Zou et al., 2010; Brandt et al., 2012), whereas CPK21 phosphorylates K^+^ outward rectifying channel GORK to promote the efflux of K^+^ ions; KAT1 and KAT2 are inactivated by CPK13 to inhibit K^+^ ion influx (Hosy et al., 2003; van Kleeff et al., 2018). In contrast, the Ca^2+^-independent pathway is associated with SnRK2.6 (Li et al., 2000), which activates SLAC1 and KUP6, the latter of which is a KUP/HAK/KT family K^+^ efflux transporter, thereby suppressing KAT1 activity *via* phosphorylation (Kwak et al., 2001; Geiger et al., 2009; Lee et al., 2009; Sato et al., 2009a; Osakabe et al., 2013). SnRK2.6 has also been shown to phosphorylate the ABA-responsive kinase substrate AKS1, which binds to the *KAT1* promoter, thereby promoting the downregulated expression of *KAT1* (Takahashi et al., 2013). Collectively, these Ca^2+^-dependent and Ca^2+^-independent pathways contribute to reductions in guard cell turgor, thereby leading to stomatal closure.

### Regulation of Dormancy and Germination

As sessile organisms, plants are particularly responsive to environmental cues with respect to the initiation of germination. Under unfavorable environmental conditions, ABA contributes to the suppression of germination by promoting seed dormancy, as revealed by the reduced seed dormancy of ABA biosynthesis and signaling-impaired mutants (Koornneef et al., 1982; Leon-Kloosterziel et al., 1996; Lefebvre et al., 2006; Nakashima et al., 2009a; Zhao et al., 2018). For example, the Raf-like MAKKKs, Raf10 and Raf11, influence seed dormancy by phosphorylating SnRK2s and ABRE-binding factors (ABFs; Lee et al., 2015; Nguyen et al., 2019). ABA can also influence seed germination by modifying hormonal balance. In contrast to ABA, gibberellic acid (GA) is a phytohormone that promotes seed germination (Debeaujon and Koornneef, 2000; Ogawa et al., 2003), and the activities of these two hormones antagonistically regulate seed dormancy and germination in higher plants (Weiss and Ori, 2007; Golldack et al., 2013). Notably, compared with wild-type plants, ABA-deficient mutants are characterized by higher levels of GA, and it has accordingly been suggested that ABA plays a role in regulating the GA metabolic pathway (Seo et al., 2006). As a key repressor of GA signaling, REPRESSOR OF GA-LIKE 2 (RGL2) plays a negative role in seed germination, as indicated by the loss-of-function mutant *rgl2*, in which ABA concentrations are reduced and germination is promoted (Piskurewicz et al., 2008; Lee et al., 2010). RGL2 also promotes *ABI5* expression levels, thereby inducing the ABA-mediated suppression of germination (Liu et al., 2016). Exogenous ABA activates the expression of *RGL2* and *ABI5* (Piskurewicz et al., 2008), and thus RGL2 is believed to modulate the balance between ABA and GA contents during seed germination.

### Regulation of Drought-Responsive Gene Expression

On exposure to drought stress, ABA induces the expression of several genes in mature plants that play roles in the drought stress response. Representative ABA-responsive marker genes in this context include *RESPONSIVE TO DESICCATION 29B (RD29B), RESPONSIVE TO ABA18 (RAB18), LATE EMBRYOGENESIS ABUNDANT 1 (EM1),* and *EM6*, the expression of which is induced under conditions of water limitation (Yamaguchi-Shinozaki and Shinozaki, 1994; Mantyla et al., 1995; Uno et al., 2000; Carles et al., 2002; Umezawa et al., 2006; Wang et al., 2018b). The expression of these genes is regulated by a number ABA-inducible transcription factors, including ABA-responsive element (ABRE)-binding proteins (AREBs) and ABFs, which contribute to the regulation of ABA-dependent gene expression under drought stress conditions (Nakashima et al., 2009b; Yoshida et al., 2015). It has also been established that a number drought stress-induced genes are also responsive to exogenous ABA, which is mediated *via* promoter region ABREs (Nakashima et al., 2009b; Fujita et al., 2011). For example, plants overexpressing (OX) the *AREB1/ABF2, AREB2/ABF4, ABF1,* and *ABF3* genes are characterized by enhanced ABA sensitivity and drought tolerance (Kang et al., 2002; Fujita et al., 2005). In contrast, *areb1*, *areb2*, and *abf3* mutants show reduced drought tolerance and ABA sensitivity (Yoshida et al., 2010). The transcripts of other transcription factors, such as *AtMYC2* and *AtMYB2*, have been found to accumulate in response to exogenous ABA, and in *AtMYC2*-OX and *AtMYB2*-OX plants, the ABA-inducible gene *RESPONSIVE TO DESICCATION 22 (RD22)* is upregulated, leading to ABA-sensitive and drought-tolerant phenotypes (Abe et al., 2003). Similarly, expression of the NAC transcription factor *RD26* is induced by drought and ABA treatment, with *RD26-*OX plants being characterized by hypersensitivity to exogenous ABA, whereas contrastingly, *RD26* dominant repressor plants exhibit an ABA-insensitive phenotype (Fujita et al., 2004). Collectively, these findings thus indicate that ABA induces ABA-responsive transcription factors, which in turn regulate the expression of ABA-responsive genes, thereby enhancing drought stress tolerance.

### Regulation of Leaf Senescence

Exogenous ABA treatment has been widely demonstrated to induce leaf senescence (Gepstein and Thimann, 1980; Pourtau et al., 2004; Raab et al., 2009; Lee et al., 2011). Moreover, it has been observed that as leaves senesce, there is a corresponding increase in endogenous ABA levels (Gepstein and Thimann, 1980; Leon-Kloosterziel et al., 1996; Cheng et al., 2002; Breeze et al., 2011; Yang et al., 2014). In terms of gene expression, ABA signaling-associated genes have been found to be upregulated during leaf senescence and the expression of various senescence-associated genes can also be induced by the application of exogenous ABA (Tan et al., 2003; Parkash et al., 2014). An important factor associated with the induction of leaf senescence is chlorophyll degradation, which is mediated *via* genes such as *STAY-GREEN 1 (SGR1)*, *NON-YELLOW COLORING 1 (NYC1), PHEOPHYYTINASE (PPH),* and *PHEIDE a OXYGENASE (PaO)* (Pruzinska et al., 2005; Ren et al., 2007; Hortensteiner, 2009; Sato et al., 2009b; Schelbert et al., 2009). In this regard, *Arabidopsis thaliana* NAC-LIKE, ACTIVATED BY AP3/PI (AtNAP) has been found to bind to the promoter of *ABSCISIC ALDEHYDE OXIDASE 3 (AAO3)* to enhance *AAO3* transcription and ABA production, and this accumulation of ABA contributes to the induction of chlorophyll degradation-associated genes (Yang et al., 2014). ABA core components have also been established to be involved in chlorophyll degradation. For example, SnRK2s regulate the bZIP transcription factors ABI5 and ABF2/3/4, which directly activate *NYC1*, *PAO*, and *SENESCENCE-ASSOCIATED GENE 12* (*SAG12*), which are considered senescence marker genes (Gao et al., 2016; Zhao et al., 2016b).

## ABA Core Components

Under abiotic stress conditions, ABA mediates responses *via* a core signaling pathway. As ABA receptors, PYR1 and PYLs were initially identified based on the characterization of ABA-insensitive mutants, whereas RCARs were identified *via* yeast two-hybrid screens with clade A PP2Cs (Ma et al., 2009; Park et al., 2009). As positive regulators of ABA signaling, PYRs, PYLs, and RCARs (Ma et al., 2009; Park et al., 2009; Santiago et al., 2009; Gonzalez-Guzman et al., 2012; Antoni et al., 2013) are characterized by star-related lipid-transfer domains that contain a conserved ligand-binding pocket and are localized to the nucleus, cytoplasm, and plasma membrane (Iyer et al., 2001; McConnell et al., 2001; Radauer et al., 2008). *Arabidopsis* also expresses a group of 13 genes that are similar to *Pyr1,* namely, *Pyl1* to *Pyl13* (*PYR1-Like*; Fujii et al., 2009; Li et al., 2013; Zhao et al., 2013), among which, *Pyr1/Pyl1/pyl2/pyl4* mutant alleles show an ABA insensitive phenotype in terms of seed germination and root growth with diminished ABA-responsive gene expression and SnRK2.6 kinase activity (Park et al., 2009). Under normal conditions, PP2Cs negatively regulate ABA signaling by interacting with SnRK2s and inhibiting kinase activity, whereas under stress conditions, PYR1 and PYLs interact with group A PP2Cs, leading to the release and activation of SnRK2s. PYLs have different binding properties with respect to ABA and PP2Cs (Szostkiewicz et al., 2010; Hao et al., 2011; Antoni et al., 2012; Li et al., 2013; Zhao et al., 2013; Tischer et al., 2017), with two discrete binding types being identified, namely ABA-enhanced and ABA-dependent. In the former PYLs, including PYL4-6 and PYL8-10, bind with PP2Cs, whereas in the latter, dimeric PYLs, including PYR1 and PYL1-2, bind with PP2Cs (Hao et al., 2011).

In *Arabidopsis*, six of the nine identified clade A PP2Cs (ABI1, ABI2, HAB1, HAB2, AHG1, and PP2CA) function as ABA negative regulators (Merlot et al., 2001; Kuhn et al., 2006; Robert et al., 2006; Saez et al., 2006; Yoshida et al., 2006b; Nishimura et al., 2007; Rubio et al., 2009). Under normal conditions, PP2Cs bind to SnRK2s, thereby suppressing stress signal transmission, and thus facilitating appropriate plant growth. PP2Cs interact with C-terminal subdomain II of SnRK2s in an ABA-independent manner (Bhaskara et al., 2012) and repress ABA signaling *via* interaction with and dephosphorylation of SnRK2s (Mustilli et al., 2002; Yoshida et al., 2006a; Fujii et al., 2007; Lee et al., 2009; Umezawa et al., 2009; Vlad et al., 2009). In the presence of ABA, the different PP2Cs interact with specific ABA receptors and release SnRK2s, thereby inducing downstream signal transduction. Released subclass III SnRK2s are autophosphorylated and activated, with their activity being enhanced *via* phosphorylation by other protein kinases, including Raf kinases (Cai et al., 2014; Nguyen et al., 2019). In turn, these activated SnRK2s phosphorylate downstream transcription factors, including AREB and ABFs (Kobayashi et al., 2005; Furihata et al., 2006; Sirichandra et al., 2010).

## Post-translational Modification of ABA Components

### Ubiquitination and Deubiquitination

**Figure 1:**
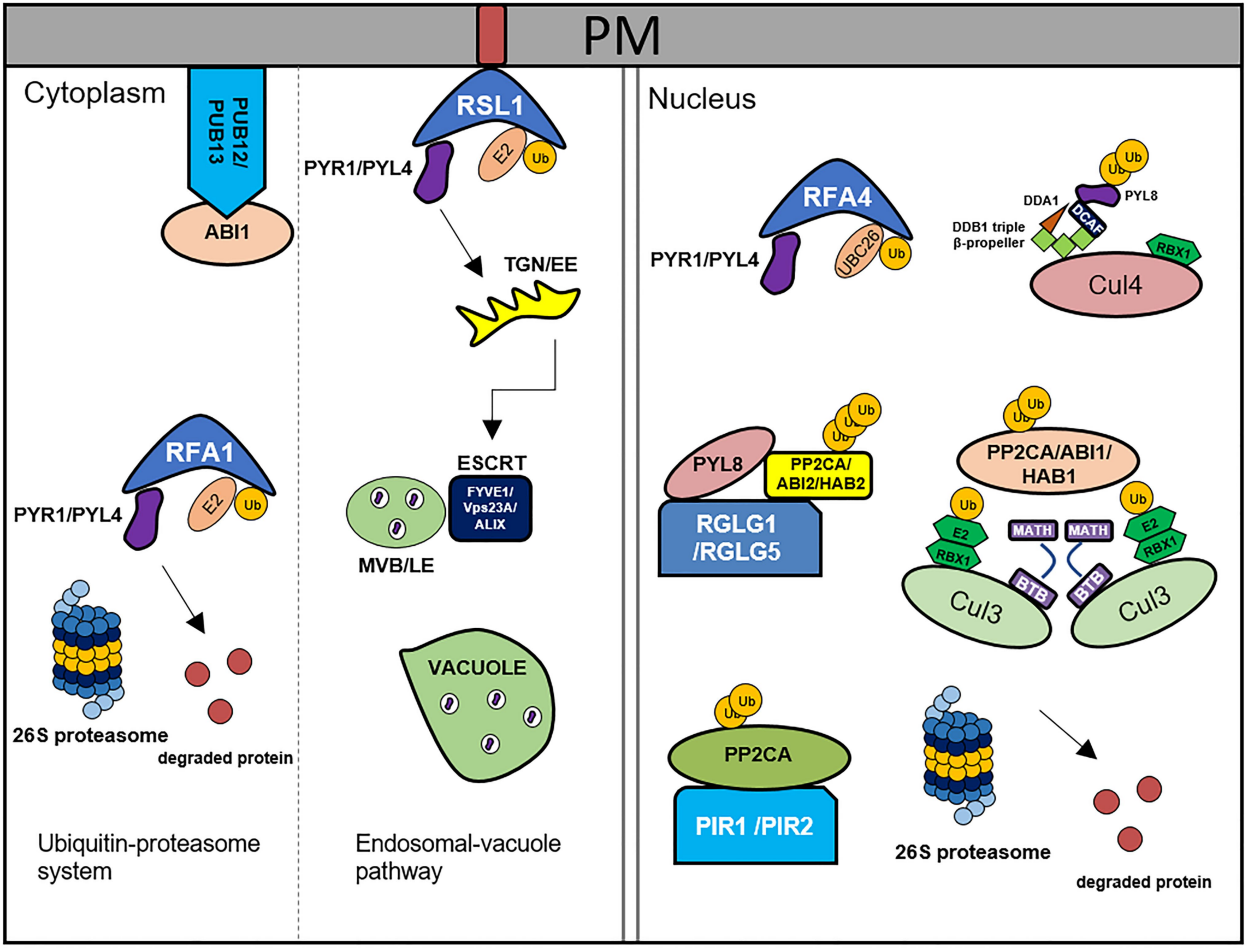
Degradation of ABA core components by the 26S ubiquitin-proteasome system and endosomal-vacuole pathway. PYR1/PYLs are degraded by RSL1, RFA1/RFA4, and CRL4^DDA1^ through ubiquitin-proteasome system. RSL1 ubiquitinates PYR1/PYL4 on the plasma membrane (PM), after that ubiquitinated substrates are degraded by ESCRT components, FIVE1/VPS23A/ALIX *via* endosomal-vacuole pathway. RFA1- and RFA4/CRL4^DDA1^-mediated ubiquitination processes occur in the cytosol and nucleus, respectively. PP2Cs are degraded by PUB12/13, CRL3^BPM3/5(BTB-MATH)^, PIR1/2, and RGLG1/5 through 26S proteasome. PUB12/13 degrade ABI1 by ubiquitination in the PM. RGLG1/5 ubiquitinate PP2CA/ABI2/HAB2, CRL3^BPM3/5(BTB-MATH)^ ubiquitinate PP2CA/ABI1/HAB1, and PIR1/2 ubiquitinates PP2CA in the nucleus. This figure has been modified from Coego et al. (2021).

The core components of ABA signaling are modulated by different types of post-translational modification, among which, ubiquitination influences the responses to environmental factors. Ubiquitination is mediated by the activity of a series of enzymes, including ubiquitin-activating enzyme E1, ubiquitin-conjugating enzyme E2, and ubiquitin-protein ligase E3, and the substrate proteins thus ubiquitinated are subsequently degraded by 26S proteasome proteolysis (Stone, 2019). ABA components are regulated by E3 ligase complexes *via* successional steps (Figure 1). These complexes are classified into four groups, namely, really interesting new gene (RING), cullin-RING ligase (CRLs), U-box, and homologous to E6-AP carboxyl terminus (HECT; Vierstra, 2009), among which, RING, CRLs, and U-box E3 ligases are involved in ABA signaling.

Deubiquitination is catalyzed by the ubiquitin deconjugating enzymes (Chung and Baek, 1999). In contrast to E3 ligases, members of the deubiquitinase superfamily (DUB) proteins cleave ubiquitin from target proteins *via* the deconjugation or degradation of ubiquitin (Amerik and Hochstrasser, 2004). These processes contribute to enhancing target protein stability and activity *in vivo* (Taya et al., 1999; Lim et al., 2021b). There are five superfamilies among the DUBs, namely, ubiquitin-specific-processing proteases (UBPs), ubiquitin carboxy-terminal (UCH) proteases, the ovarian tumor proteases (OTUs), the Machado-Joseph disease protein domain proteases (MJDs), and JAB1/MPN^+^/MOV34 (JAMM) proteases. UBPs, UCHs, OTUs, and MJDs are cysteine proteases, whereas JAMMs are zinc metalloisopeptidases (Nijman et al., 2005). Of these five families, the UBPs are well studied in plants, and have been shown to play important roles in cellular processes, including signal transduction, protein degradation, and gene regulation (Yan et al., 2000; Liu et al., 2008).

#### Ubiquitination of ABA Receptors

CRL E3 ligase complexes and RING-type E3 ligases degrade PYR/PYLs *via* the 26S proteasome, and yeast two-hybrid screening has revealed that in this pathway, Cullin 4 (CUL4)/De-etiolated 1 (DET1)-damaged DNA binding protein 1 (DDB1)-associated 1 (DDA1) E3 ligase interacts with PYL4, PYL8, and PYL9 (Irigoyen et al., 2014). Furthermore, DDA1 promotes PYL8 protein degradation *via* the 26S proteasome and reduces ABA sensitivity in *DDA1* overexpression mutants (Irigoyen et al., 2014), whereas in MG132-treated plants, accumulated PYL8 is polyubiquitinated and DDA1 promotes polyubiquitination, but ABA opposes this effect (Irigoyen et al., 2014). These findings provide evidence to indicate that ABA protects PYL8 from CRL4^DDA1^-mediated degradation, either by affecting the CDD-DDA1 complex or by promoting the formation of degradation-resistant PYL8-PP2C complexes (Irigoyen et al., 2014).

Ring finger of seed longevity 1 (RSL1), a member of the RBR-type E3 ligase family (Marin, 2010; Callis, 2014) interacts with PYR1 and PYL4 in the plasma membrane and subsequently polyubiquitinates these to promote their degradation *via* the 26S proteasome. Phenotypic analysis has revealed that RNA interference mutants of *RSL1* are characterized by an ABA-hypersensitive phenotype, whereas an *RSL1* overexpression line was found to show reduced ABA sensitivity during seed germination and early seedling growth (Bueso et al., 2014). These observations indicate that RSL1 plays a negative role in ABA signaling *via* the degradation of ABA receptors (Bueso et al., 2014). In the same gene family as *RSL1,* the RING finger ABA-related 1–9 (RFA1 to RFA9) proteins are similarly associated with ABA receptor degradation, among which, RFA1 and RFA4 are localized in the nucleus and promote the nuclear degradation of PYR1 and PYL4 *via* activity of the nuclear E2 enzyme UBC26 (Fernandez et al., 2020). Compared with wild-type plants, PYR1 and PYL4 were observed to show higher expression in an *RFA1*/*RFA4* double knock-out mutant (Fernandez et al., 2020). The endosomal trafficking pathway functions as an important route for the modification of membrane proteins *via* the endosomal sorting complex required for transport (ESCRT) machinery. Ubiquitinated PYL4 is localized in the plasma membrane and degraded *via* a vacuolar degradation pathway mediated by the ESCRT components, in which ESCRT-I components FIVE1, VPS23A, and ALIX promote PYL4 degradation. Knock-down mutations of these ESCRT components are characterized a reduction in the degradation of PYL4 and enhanced ABA responses (Belda-Palazon et al., 2016; Yu et al., 2016; Garcia-Leon et al., 2019).

#### Ubiquitination of Clade A PP2Cs

Plant U-box type E3 ubiquitin ligase (PUB)12/13, Ring domain ligase (RGLG)1/5, CUL3-RING-based E3 ligases, and other E3 ligases collectively play roles in the proteolytic degradation of PP2Cs. PUB12/13 interact with ABI1 and thereby promote the ubiquitination of ABI1 in response to exogenous ABA, which induces the production of the dimeric form of PYR1 or monomeric forms of PYL4/PYL9 (Kong et al., 2015). A *pub12 pub13* double mutant was found to be characterized by a high accumulation of ABI1 and an ABA-insensitive phenotype, the latter of which was recovered following the crossing of double mutant plants with those containing an *abi1-3* mutation. These findings thus indicate that PUB12 and PUB13 play roles in controlling ABI1 stability (Kong et al., 2015). RGLG1/5 E3 ligases contribute to the degradation of PP2CA. *In vivo* analysis has revealed that RGLG1 interacts with PP2CA, which is enhanced by ABA, and promotes ubiquitination. *In vitro*, however, it has been observed that RGLG1 ubiquitinates PP2CA in the absence of either ABA or PYL4 (Wu et al., 2016). In contrast to PP2CA, which is nuclear localized, myristoylated RGLG1 is localized in the plasma membrane. ABA prevents the myristoylation of RGLG1 and induces cycloheximide-insensitive translocation to the nucleus (Belda-Palazon et al., 2019). Thus, RGLG1 plays a positive role in the ABA response by repressing PP2CA (Wu et al., 2016). PP2CA has also been demonstrated to interact with BTB/POZ and MATH domain proteins (BPMs) in plants, based on co-immunoprecipitation analysis performed in conjunction with liquid chromatography–tandem mass spectrometry. Compared with wild-type plants, those overexpressing *BPM3* and *BPM5* were found to accumulate lower amounts of PP2CA protein following ABA treatment. Contrastingly, in response to ABA treatment, the levels of PP2CA accumulated in plants harboring a *bpm3 bpm5* double knock mutation were observed to be higher than those in the wild-type plants. Consistently, *BPM3* and *BPM5* overexpressing plants are characterized by enhanced ABA sensitivity, whereas the *bpm3 bpm5* mutant shows reduced ABA sensitivity. Consequently, CRL3^BPM^ complexes appear to play vital roles in ABA responses and signaling (Julian et al., 2019).

By interacting with UBC27, AIRP3 contributes to ABI1 degradation *via* E3 ligase activity. The UBC27-AIRP3 ubiquitination complex modulates ABA responses by enhancing ABI1 protein stability. In *ubc27* and *airp3* mutant plants, the rate of ABI1 degradation is reduced, and with respect to cotyledon greening and stomatal movement, *ubc27* and *airp3* mutant plants are characterized by ABA-insensitive phenotypes (Pan et al., 2020). Among other RING proteins, PP2CA interacting ring finger protein (PIR)1 and PIR2 have been shown to interact with PP2CA, both *in vitro* and *in vivo*, thereby promoting its ubiquitination. *pir1 pir2* double knock-out mutants were found to be characterized by higher PP2CA protein levels, thus indicating that PIR1 and PIR2 degrade PP2CA *via* the 26S proteasome (Baek et al., 2019b).

#### Regulation of SnRK2s Stability by E3 Ligases

Although E3 ligases have been established to contribute indirectly to the stability of SnRK2s, there has as yet been no conclusive evidence to indicate that E3 ligases directly target SnRK2s. To date, only AtPP2-B11, a component of the SKP1/Cullin/F-box complex, has been shown to interact directly with SnRK2.3, with cell-free degradation assays performed using *AtPP2-B11-OE* and *amiR-AtPP2-B11* cell extracts indicating that AtPP2-B11 negatively influences SnRK2.3 stability. However, there is no evidence to indicate that AtPP2-B11 directly ubiquitinates SnRK2.3 (Cheng et al., 2017). In addition, the E3-ubiquitin ligase HOS15 has been found to interact with SnRK2.6, thereby influencing its stability, and compared with wild-type plants, those harboring a *hos15-2* mutation were found to accumulate higher levels of SnRK2.6 (Ali et al., 2019). However, in common with AtPP2-B11, the mechanisms whereby HOS15 regulates SnRK2 stability have yet to be established.

#### Modulation of ABA Signaling by Ubiquitin-Specific Processing Proteases

With respect to ABA signaling, plant tolerance to high salinity and drought stresses is modulated by UBP24 *via* the regulation of stomatal closure. Knock-out of the *UBP24* gene confers an ABA-hypersensitive phenotype during seedling growth, although contrastingly it reduces ABA sensitivity in the guard cells of mature plants. The latter effect results in a drought-sensitive phenotype, owing to a higher water loss compared with wild-type plants (Zhao et al., 2016a). In pepper plants, CaUBP12 has been observed to suppress the degradation of CaSnRK2.6 (Arabidopsis OST1 homolog; Lim et al., 2021b). CaUBP12 interacts with CaSnRK2.6 both *in vitro* and *in vivo*, and cell-free degradation analysis has revealed higher levels of CaSnRK2.6 degradation when incubated with crude extracts of the *CaUBP12* knock-down pepper plants, although lower levels in the presence of crude extracts of *CaUBP12* overexpressing *Arabidopsis* and tobacco plants (Lim et al., 2021a,b). These findings thus provide evidence to indicate that deubiquitinase-mediated post-transcriptional modification is implicated in the modulation of ABA signaling.

### Phosphorylation of ABA Core Components

**Figure 2:**
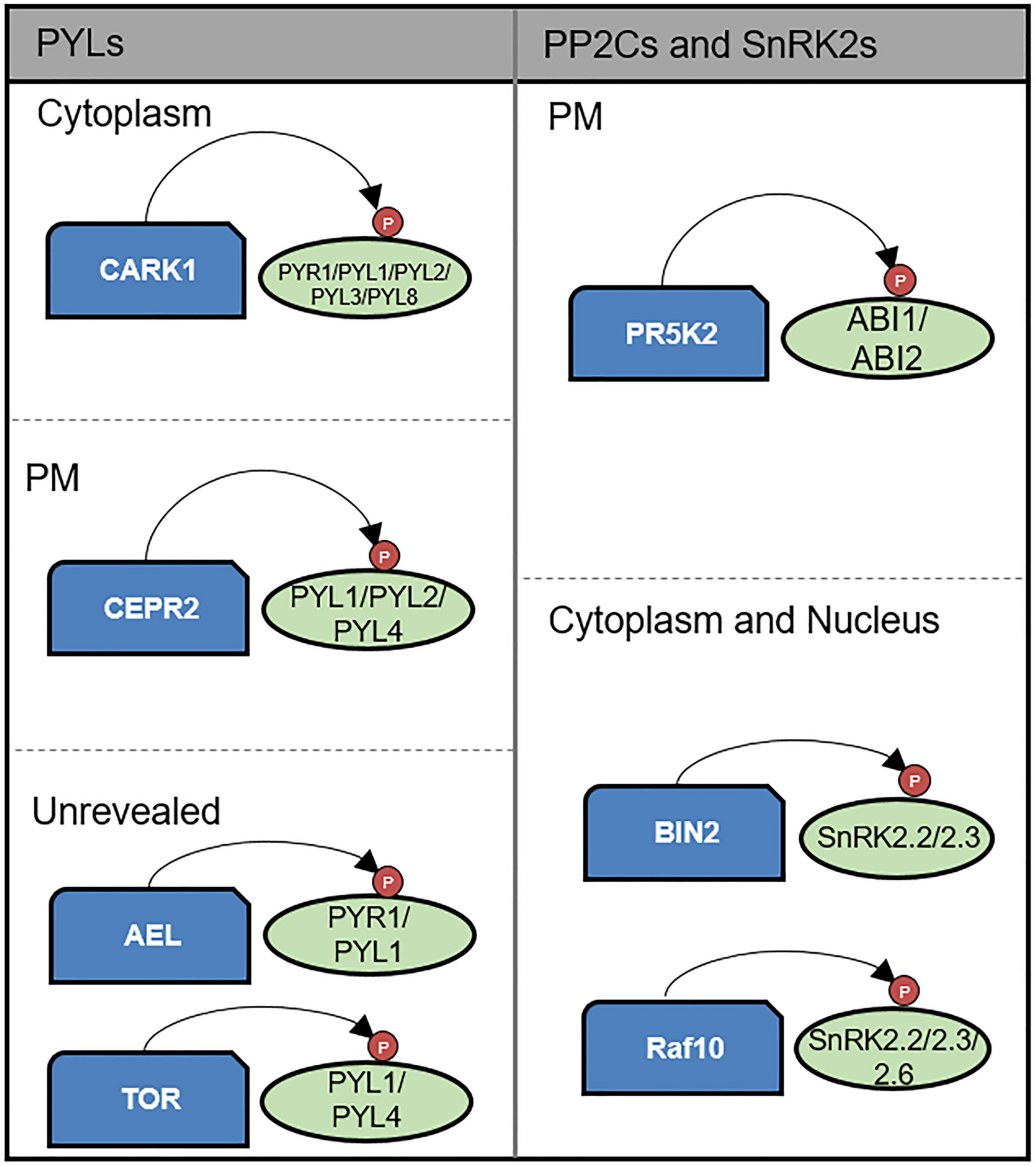
Kinase-mediated post-translational modification of core components of the ABA signaling pathway. CARK1, CEPR2, TOR kinase, and AEL phosphorylate PYLs. CARK1 positively regulates ABA signaling by enhancing PYLs activity on the cytoplasm. In contrast, CEPR2 and AEL negatively regulate ABA signaling by promoting degradation of PYLs. TOR kinase also plays negative role in ABA signaling by preventing PYL activation. CEPR2 acts on PM. ABI1 and ABI2 are phosphorylated and enhanced in their phosphatase activity by PR5K2 on the PM. BIN2 and RAF10 phosphorylate SnRK2s enhancing their kinase activity on the cytoplasm and nucleus.

In response to the perception of stress, ABA signaling is regulated *via* the phosphorylation activity of a diverse range of kinases particularly with respect to ABA-induced responses under drought stress (Zhu, 2016). In the ABA core signaling pathway, SnRK2s phosphorylate downstream transcription factors (Merlot et al., 2001; Yoshida et al., 2002, 2006b; Saez et al., 2004, 2006; Kuhn et al., 2006; Robert et al., 2006; Rubio et al., 2009), whereas other kinases contribute to the phosphorylation of ABA receptor PYR/PYLs, PP2Cs, SnRK2s, and downstream transcription factors (Figure 2; Cai et al., 2014; Chen et al., 2018; Wang et al., 2018a; Zhang et al., 2018; Baek et al., 2019a; Li et al., 2019; Nguyen et al., 2019; Yu et al., 2019).

#### Phosphorylation of ABA Receptors

Several studies have provided evidence indicating the phosphorylation of ABA receptors. For example, TOR kinase, *Arabidopsis* Early flowering 1 (EL1)-like casein kinase (AEL), C-terminally encoded peptide receptor 2 (CEPR2), and cytosolic ABA receptor kinase 1 (CARK1) have been established to phosphorylate PYLs (Chen et al., 2018; Wang et al., 2018a; Zhang et al., 2018; Li et al., 2019; Yu et al., 2019). Among these, TOR kinase inactivates PYL1 and PYL4 by phosphorylating the Ser119 and Ser114 residues, respectively. Phosphomimic mutants of PYL1 and PYL4 are characterized by an inability to inhibit ABI1 phosphatase activity. Moreover, ABA has been shown to inhibit TOR kinase activity, thereby enhancing the ABA response. Thus, these observations indicate that TOR kinase functions as a negative regulator of ABA signaling by phosphorylating PYL1 and PYL4 (Wang et al., 2018a). AEL phosphorylates PYR1 and PYL1 at residues Ser109/152 and Ser136/182, respectively, and in doing so, promotes PYR1 and PYL1 degradation. Compared with wild-type plants, PYR1 and PYL1 proteins show greater stability in *ael* triple mutants. The ABA- and NaCl-sensitive phenotypes in knock-out mutants thus tend to indicate that AEL negatively regulates ABA signaling (Chen et al., 2018). CEPR2 has been found to interact with PYL4 in the plasma membrane, in which it phosphorylates Ser54, thus promoting an accelerated degradation of the PYL4 protein. Consistently, the levels of PYL4 protein in *CEPR2* overexpressing and *cepr2* mutant plants have been shown to be less and more stable, respectively (Yu et al., 2019). CARK1 phosphorylates PYR1 Thr78, PYL1 Thr105, PYL2 Ser81, and PYL3 Thr101, thereby enhancing ABA responses implicated in the inhibition of seed germination, root growth, and the expression of a number of ABA-responsive genes (Zhang et al., 2018; Li et al., 2019).

#### Phosphorylation of Clade A PP2Cs and Subclass III SnRK2s

PP2Cs are also phosphorylated by specific kinases. For example, ABI1 and ABI2 are phosphorylated by PR5 receptor-like kinase 2 (PR5K2), which phosphorylates the putative ABI1 phosphorylation site Ser 314 and ABI2 Ser304, thereby enhancing their respective phosphatase activities (Baek et al., 2019a). Plants overexpressing PR5K2 are characterized by ABA-insensitive and drought-sensitive phenotypes, whereas *atpr5k2* mutant plants show the opposite phenotypes. SnRK2s are phosphorylated and activated by Raf-like MAKKKs, among which RAF10 phosphorylates SnRK2s at a site within C-terminal domain II, referred to as the ABA box. *raf10 raf11* double-knock-out mutants are characterized by reduced levels of SnRK2 phosphorylation and germination is significantly delayed in the presence of ABA. In brassinosteroid (BR) signaling, BRASSINOSTEROID INSENSITIVE 2 (BIN2) plays a negative regulatory role by phosphorylating several transcription factors, which contrast with ABA signaling, wherein BIN2 plays the role of a positive regulator promoting ABA responses *via* the phosphorylation of SnRK2.2 and SnRK2.3, thus enhancing their kinase activities. These observations indicate that the modulation of stress responses in plants involves a certain degree of crosstalk between the ABA and BR signaling pathways (Cai et al., 2014).

## Conclusions and Perspectives

Abscisic acid plays vital roles in plant growth, development, and biotic/abiotic stress responses, particularly with respect to seed dormancy/germination, stomatal opening and closure, and leaf senescence. Moreover, the ABA core signaling pathway and post-translational modification of pathway components have been extensively studied. Ubiquitination, catalyzed by E3 ligases, contributes to the 26S proteasome-mediated degradation of ABA receptor PYR/PYL and clade A PP2C proteins, and numerous E3 ligases have been found to regulate the stability of these target proteins, either *via* direct or indirect interactions. However, it has yet to be conclusively determined whether E3 ligases directly ubiquitinate SnRK2 proteins, which accordingly limits our current understanding of the E3 ligase-mediated modulation of SnRK2 protein stability. The regulation of target protein stability is also modulated by deubiquitination, although in *Arabidopsis*, the mechanisms underlying the deubiquitination of ABA signaling core components remain to be ascertained. The involvement of UBPs in stress responses and other developmental processes is well characterized; however, to date there have been few studies that have examined the mechanisms associated with protein deubiquitination. To determine whether target proteins are deubiquitinated by UBPs, it will be necessary to isolate either mono- or poly-ubiquitinated proteins. The phosphorylation of target proteins also contributes to their activity and/or stability. In this regard, several kinases that phosphorylate key ABA signaling factors have been shown to regulate drought stress responses, either positively or negatively. However, only a few kinases involved in the ABA core signaling-associated regulation of drought responses have been examined, and it can be assumed that there are numerous other functional kinases that await identification. Consequently, to gain a better understanding of the post-translational modification of ABA core components, it will be necessary to conduct further studies that focus on identifying and characterizing these undiscovered proteins, which will illustrate potentially novel routes for the regulation of ABA signaling and stress responses.

## Author Contributions

JL, CL, and SL: conceptualization and writing. JL and CL: data analysis. All authors contributed to the article and approved the submitted version.

## Funding

This work was supported by a grant from the Agriculture & Technology Development (project no. PJ01652101), the National Research Foundation of Korea (NRF) grant funded by the Korea Government (MSIT; 2021R1A2C2006338), and Rural Development Administration, Republic of Korea.

## Conflict of Interest

The authors declare that the research was conducted in the absence of any commercial or financial relationships that could be construed as a potential conflict of interest.

## Publisher’s Note

All claims expressed in this article are solely those of the authors and do not necessarily represent those of their affiliated organizations, or those of the publisher, the editors and the reviewers. Any product that may be evaluated in this article, or claim that may be made by its manufacturer, is not guaranteed or endorsed by the publisher.
